# Preventive maintenance in urban public transport: the role of engine oil analysis

**DOI:** 10.1038/s41598-024-81728-w

**Published:** 2024-12-28

**Authors:** Wojciech Gołębiowski, Artur Wolak, Grzegorz Zając

**Affiliations:** 1 Department of Power Engineering and Transportation, University of Life Sciences in Lublin, Gleboka 28, 20-612 Lublin, Poland; 2 Department of Quality and Safety of Industrial Products, Cracow University of Economics, Sienkiewicza 4, 30-033 Cracow, Poland

**Keywords:** Engine oil, FTIR, XRF, Blotter spot test, Degradation, CBM, Mechanical engineering, Chemical engineering

## Abstract

Engine oil is a valuable source of information on the technical condition of the drive unit. Under the influence of many factors, including operating conditions, time, high temperature, and various types of contamination, the oil gradually degrades, which can result in serious engine damage. The subject of the article focuses on an attempt to answer the questions of how engine failure affects the degradation of engine oil and whether we can use this knowledge to detect potential problems in public transport vehicles at an early stage. The research material consisted of samples of engine oil in the SAE 10 W-40 viscosity class and data on vehicle faults obtained from the service of a public transport company. The oils come from two city buses belonging to the fleet of diesel-powered vehicles, in which critical cooling system failures were diagnosed during engine tests, excluding the vehicle from further use. The conducted studies analyzed the degree of oil degradation, which included determining the changes in kinematic viscosity at 40 °C and 100 °C via a Stabinger SVM 3001 viscometer. The physicochemical parameters of the oil, such as the degree of oxidation, nitration, total acid number (TAN), total base number (TBN), and content of impurities in the form of soot and glycol, were examined via FTIR spectroscopy. The degree of impurities and the general quality of the oil were also determined via a blotter spot test. Additionally, to determine the degree of metal abrasion and changes in the depletion of additives, elemental analysis via the HDXRF method was used. As a result of the tests carried out, potential correlations between the oil condition and the technical condition of the vehicle in real operating conditions were confirmed.

## Introduction

In 2022, the number of passenger cars on the roads of the European Union exceeded 252 million units^1^. The number of these vehicles is constantly growing annually^2^. According to the ACEA report^1^, the average age of cars in the EU is 12.3 years, meaning many vehicles are equipped with older, less efficient technologies, leading to increased environmental emissions. Poland has the highest car density per 1,000 inhabitants at 703 units. Despite the large increase in sales of electric vehicles (battery electric and plug-in hybrids) in recent years, this type of vehicle accounts for only 1.2% of the total vehicle fleet in the EU.

In the fight with decarbonization of the transport sector promoted by the European Union (EU), in addition to encouraging investments in the purchase of new vehicles using renewable energy sources, one cannot forget another important aspect of sustainable transport, which is public transport^3,4^. Each bus driving on the roads replaces 50 passenger cars, helping to relieve congestion on the road, reducing exhaust emissions and thus improving air quality^5,6^. Unfortunately, as in the case of passenger cars, the age of the bus fleet in the EU is 12.5 years. Diesel engines are the predominant power source for buses within the European Union, constituting 90.5% of the total fleet. Transport, both freight and passenger, operates under conditions of increased risk of unexpected failures, which requires ensuring the reliability of the services provided^7,8^. Given the age of the vehicle fleet, a more reliable and sustainable approach to maintenance is urgently needed to avoid breakdowns, reduce emissions, and reduce waste^9,10^.

A variety of maintenance techniques and methodologies can be employed to predict equipment failure^11,12^. The most widely used maintenance approaches include reactive, preventive, and predictive strategies^13^. Lubricant condition monitoring (LCM) has emerged as an essential tool in condition monitoring because of its ability to provide a wealth of diagnostic information through the analysis of lubricating oils. By examining the physical, chemical, and tribological properties of oil, LCM offers invaluable insights into the internal conditions of machines and equipment^14–17^. Through the detection of wear particles, contaminants, and degradation products, LCM enables early identification of potential failures, thereby facilitating proactive maintenance strategies and preventing catastrophic breakdowns^18–21^. Additionally, LCM plays a pivotal role in optimizing engine oil management, extending equipment life, and enhancing overall operational efficiency^22,23^. LCM testing consist of analyses focused on physicochemical, elemental, additive, and contaminant parameters.

Recently, engine oil condition monitoring and equipment condition prediction have become critical areas of interest in academia and industry^24–27^. Efforts are being made to proactively prevent machine failures by analyzing the condition of the lubricating oil^27–29^. Implementing such solutions in industry and transportation extends service life, reduces costs associated with unexpected downtime, and minimizes negative impact on the natural environment^30–32^.

Numerous studies support the use of real-world driving conditions data in formulating maintenance strategies based on the assessment of technical conditions^15,31,33,34^. Based on the analysis of failure log data obtained from a public transport company, it was found that for 53 buses used during the analyzed 2-year period, more than 40% of critical failures resulting in vehicle immobilization were related to problems with the cooling system. Developing an oil condition test to predict such failures would allow the company to reduce the operating costs of repairing bus stops. The investigation into the relationship between observed failures and oil condition utilized a combination of kinematic viscosity, spectrometric, elemental, and blotter spot test analyses.

This study aims to investigate the changes in engine oil parameters that occur in vehicles experiencing critical failure. Both vehicles involved suffered severe malfunctions that necessitated immobilization and extensive engine repairs. The unique nature of this study contributes to our understanding of how engine failure impacts engine oil characteristics. Additionally, it allows us to explore whether these changes are indicative of specific types of faults. By focusing on real-world cases where critical failures occurred shortly after or during routine service intervals, this research expands our knowledge of engine oil analysis.

## Materials and methods

###  Materials

In the present study, the research materials were samples of Orlen Oil Platinum Ultor Extreme engine oil with a viscosity specification according to SAE 10W-40 and quality ACEA E4, E7, and OEM MB228.5. The viscosity and quality specifications corresponded to the recommendations of the vehicle manufacturer. The data obtained from the oil datasheet and our laboratory tests regarding the described oil are presented in Table 1.

**Table 1:** Characteristics of orlen oil platinum 10W-40 engine oil.

	Physicochemical properties	Unit	Manufacturer data	Own research
Physicochemical properties	Kinematic viscosity at 40 °C	[mm^2^/s]	-	96.0
	Kinematic viscosity at 100 °C		14.2	14.4
	Viscosity index	-	155	155.3
	Total base number (TBN)	[mg KOH/g]	12	10.6
Elemental composition	Calcium (Ca)	[mg/kg]	-	4305
	Phosphor (P)		-	960
	Sulfur (S)		-	3806
	Zinc (Zn)		-	1376

The research material was collected over 12 months. Engine oil was collected from two Autosan Sancity M12LF buses (2414 and 2378) belonging to the fleet of public transport vehicles. The buses were purchased in the years 2011–2013. These are low-floor city buses designed to carry 96–110 passengers. They are powered by Cursor diesel engines model F2BE3682B with a displacement of 7800 cm^3^. The oil pan in the tested vehicles holds 22 L of lubricating oil. The oil samples included a sample of new oil and three samples of used oil taken at different mileage points during a single oil change interval (coded as I, II and III sampling period). The vehicle manufacturer recommends changing the engine oil every 60,000 km. Oil sampling was conducted at approximately the same time and after specific mileage intervals. Deviations from the planned sampling intervals were due to the continuous operation of the buses and the availability of the vehicles at the service yard. The first sampling occurred after approximately 15,000 km, the second at the midpoint of the interval (around 30,000 km), and the final sampling took place at the time of the engine oil change. Both vehicles tested had their oils changed later than recommended by the manufacturer. One vehicle exceeded the recommended interval by 6,000 km, whereas the other exceeded it by 10,000 km. Periodic replenishment of fresh oil was conducted throughout the experiment, with each refill being recorded to evaluate its impact on the degradation of engine oil quality. The characteristics of the samples collected are summarized in Table 2.

**Table 2:** Characteristics of engine oil samples.

Sample information section						Top-ups section	
No.	Sample code	Bus model	Date of sample collection	Bus mileage [km]	Mileage since last oil change [km]	Quantity [liter]	Oil consumption per 100 km [liter]
1	2414_I	Autosan Sancity M12LF	25.10.2022	653,913	15,693	12	0.08
2	2414_II	Autosan Sancity M12LF	09.02.2023	675,751	37,535	18	0.09
3	2414_III	Autosan Sancity M12LF	02.08.2023	704,454	66,238	35 (including 7, in the last month of oil change)	0.12
4	2378_I	Autosan Sancity M12LF	21.11.2022	820,550	16,421	12	0.07
5	2378_II	Autosan Sancity M12LF	10.02.2023	835,826	31,697	16	0.11
6	2378_III	Autosan Sancity M12LF	22.09.2023	874,729	70,600	54 (including 12, in the last month of oil change)	0.14

### Data from the company vehicle’s service

**Fig. 1:**
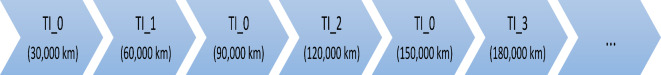
Planned maintenance—a cycle of service activities in the company.

The public transportation company employs a preventive maintenance model based on scheduled maintenance. Regular maintenance plans are implemented to minimize the risk of asset failure by conducting routine maintenance tasks at predetermined intervals. In this specific case, the maintenance plans are categorized into four types of maintenance sets, carried out by maintenance personnel. The maintenance cycle is illustrated in Fig. 1.

“TI” is an abbreviation of “Technical Inspection”. The plan marked as TI_0 includes maintenance activities performed regularly every 30,000 km, including checking lubrication points, the brake system, necessary bus equipment, electrical installations, compressed air system, washing the engine, and checking and replacing air filter inserts.

The plans marked as TI_1, TI_2, and TI_3 are repetitions of the maintenance activities included in plan TI_0, plus the performance of additional activities. In all cases of plans TI_1, TI_2, and TI_3, the engine oil and filters in the vehicles are replaced.

In the case of the TI_1 plan, additional activities performed by the service staff every 60,000 km include checking the suspension, steering system, air conditioning compressor, gearbox oil, replacing the fuel filter, and checking the engine tightness (oil, coolant, fuel).

In the case of the TI_2 plan, the activities included in the TI_0 and TI_1 plans are performed. Additionally, every 120,000 km, the brake lines are checked, among other things. The oil in the axle is changed, and the oil filter in the automatic transmission and the oil in the gearbox are replaced.

The TI_3 plan includes the activities included in the TI_0, TI_1, and TI_2 plans. For every 180,000 km of vehicle mileage, the oil filter insert and the power steering oil are replaced. Additionally, the engine valve levers are adjusted.

In addition to the above-mentioned planned maintenance cycles, service technicians regularly repair buses based on information obtained from fault reports provided to them by drivers. Tables 3 and 4 present a summary of engine failures in the tested buses during the analyzed sampling periods during one oil interval.

**Table 3:** Register of unplanned repairs of tested buses during operation (bus no. 2378).

Bus no.	Overall bus mileage [km]	Mileage on oil [km]	Date of sampling	No.	Failure description	Repair description	Number of events in database	Repair time [hours]
2378	820,550	16,421	21.11.2022	1	Critical failure – coolant in fuel	Fuel supply system repair	1	86
				2	Lack of heating	Heating – repair	6	8.5
				3	Coolant leakage	Cooling system – sealing/coolant adding	3	6
				4	Engine oil leakage	Engine – sealing	2	6
				5	Check engine – hot coolant	Cooling system – repair	1	1
				Total			13	107.5
	835,826	31,697	10.02.2023	1	Engine oil leakage	Engine – sealing	6	23
				2	Coolant leakage	Cooling system – sealing/coolant adding	2	11
				3	Lack of heating	Heating – repair	5	11
				4	Check engine(red) – hot coolant	Cooling system – repair	1	2
				Total			14	47
	874,729	70,600	22.09.2023	1	Coolant leakage	cooling system – repair	7	23
				2	Air conditioning - failure	air conditioning – repair	7	10.5
				3	Lack of heating	heating – repair	4	16
				4	Engine heating	compressed air system – repair	3	16
				5	Engine heating	water cooler - replacement	1	28
				6	Check engine – A/C failure	A/C – repair	1	2
				7	Engine oil leakage	engine – sealing	1	1
				Total			24	96.5
	Failures after oil change (1 month period)			1	Engine heating	Cooling system – repair	3	6
				2	Check engine (orange) –	Engine – oil pressure check	1	0.5
				3	Engine oil leakage	Engine - sealing	6	14
				4	Coolant leakage	Cooling system – repair	1	7
				5	Fluid leak in central heating	Circulation pump – replacement	1	2.5
				6	Critical failure – coolant in fuel	Fuel supply system repair	1	55
				Total			13	85

**Table 4:** Register of unplanned repairs of tested buses during operation (bus no. 2414).

Bus no.	Overall bus mileage [km]	Mileage on oil [km]	Date of sampling	No.	Failure description	Repair description	Number of events in database	Repair time [hours]
2414	653,913	15,693	25.10.2022	1	Engine heating − 110 °C	Cooling system – repair	4	12
				2	Coolant leakage	Cooling system – sealing/coolant adding	3	4
				3	Lack of heating	Heating - repair	3	3
				4	Engine oil leakage	Engine – sealing	1	2
				5	Check engine indicator	Engine electrical installation – repair	2	1.5
				6	Fluid leak in central heating	Central heating head - repair	1	2
				Total			14	24.5
	675,751	37,535	09.02.2023	1	Coolant leakage	Cooling system – sealing/coolant adding	12	22
				2	Check engine indicator	Engine electrical installation – repair	2	1
				3	Engine oil leakage	Engine – sealing	1	1
				4	Fuel leakage	Fuel supply system – sealing	1	2
				5	Lack of heating	Heating - repair	1	2
				6	Fluid leak in central heating	Central heating head - repair	1	4
				Total			18	32
	704,454	66,238	02.08.2023	1	Coolant leakage	Cooling system – sealing/coolant adding	21	23
				2	Check engine indicator	Engine electrical installation – repair	2	1
				3	Engine oil leakage	Engine – sealing	1	1
				4	One unit injector not working	Injector – replacement	1	4
				5	Lack of heating	Heating - repair	3	6
				6	Indicator – dirty air filter	Engine – checking	3	3
				7	A/C compressor failure	A/C repair	2	6
				8	Coolant leakage from heater	Heater - replacement	1	4
				Total			34	48
	Failures after oil change (3 months period)			1	Coolant leakage	Cooling system – sealing/coolant adding	17	16
				2	A/C failure	A/C repair	2	2
				3	Lack of heating	Critical failure –heating repair	6	23
				4	Check engine indicator	Engine electrical installation – repair	5	3.5
				5	Engine oil leakage	Engine – sealing	1	2
				6	Coolant leakage from water pump	Water pump - replacement	1	4
				Total			32	50.5

In the case of both vehicles tested, critical failures occurred, resulting in the need to immobilize the vehicle and carry out thorough engine repair. Additionally, in the case of the bus marked 2378, the first period of taking an oil sample was the period after the replacement of the engine head. In both cases, the cause was a faulty cooling system, resulting in leaks of coolant into the fuel. In addition, in bus 2414, the unit injectors failed, and there were frequent oil leaks.

In summary, the fault descriptions indicate that both cases involve cooling system failures. A potential cause is the presence of coolant or water in the oil, which can be diagnosed using FTIR spectrometric analysis and/or elemental analysis.

Another possibility is damage caused by oil overheating. Both buses experienced engine overheating, likely affecting the oil. Elevated temperatures could accelerate oxidation and degrade additives more rapidly.

A third option is oil loss and subsequent lubrication deterioration, although one bus (2378) displayed an oil warning light and low oil level. While the orange light suggested low levels rather than pressure issues, overheating oil could also contribute. Autosan Sancity M12LF buses have relatively small oil pans, which can lead to lower oil levels and reduced heat dissipation.

###  Methods

In order to check whether the engine oil analyses performed can indicate potential engine failures before they occur, a series of laboratory tests were conducted on the physicochemical properties of the oil. Additionally, changes in the additives and the engine wear were determined based on elemental analysis.

The kinematic viscosity of the tested samples at 40 °C and 100 °C was determined using an SVM Stabinger model 3001 viscometer (Anton Paar GmbH, Graz Austria). The data were analyzed to assess the direction and magnitude of viscosity changes, according to ASTM D7042^35^.

FTIR analysis was performed via a Thermo Nicolet IS10 instrument (Thermo Fisher Scientific, Massachusetts, USA) to evaluate oxidation, nitration, and soot content and additive degradation in engine oils, according to ASTM E2412^36^. The total acid number (TAN), total base number (TBN), and glycol content were measured via an ERASPEC OIL mid-infrared FTIR spectrometer (Eralytics GmbH, Austria) according to the same ASTM E2412 standard. In both cases, a comparative analysis of the FTIR spectra was performed to evaluate various parameters of fresh and used engine oils. The intensity of individual bands in used oils was determined indirectly by subtracting the spectrum of fresh oil from that of used oil, which resulted in difference spectra.

To evaluate oxidation, the absorption band around 1720 cm^−1^ was used, while nitration was analyzed at approximately 1630 cm^−1^. The soot content was assessed in two characteristic bands: around 2000 cm^−1^ and 3980 cm^−1^. A method of measuring band intensity was used, where both the height and area under the absorption band were evaluated, allowing for a quantitative assessment of chemical changes in the oil. Contaminants such as water and fuel were evaluated in the 3000–3600 cm^−1^ range, where the area of the absorption band was analyzed, allowing for a quantitative assessment of the level of these contaminants in the engine oil. The spectra were subjected to automatic baseline correction, which allowed for the elimination of possible interference resulting from baseline unevenness and improved the accuracy of the analysis of absorption band intensities. All measurements were performed in accordance with ASTM E2412, ensuring consistency and accuracy of the assessment.

The TBN and TAN parameter values were calculated by comparing the measured spectrum with a database of spectra where the parameter is defined by an appropriate reference method. Using the spectra in the database, the ERASPEC Oil device builds a multiple linear regression (MLR) model for each parameter and calculates the property parameters. The number of spectra in the database must contain at least 2 *N* + 1 spectra, where N is the number of input parameters for the MLR. The minimum number of spectra in the database for these parameters is 7, in our case, there were 57.

Glycol content was defined by the maximum absorbance within the spectral range between 1069 cm^− 1^ and 1098 cm^− 1^. The reference point for the selected peak was a baseline segment passing through two points with the lowest value, within the range defined by points P1 (1098 cm^− 1^ and 1110 cm^− 1^) and P2 (1051 cm^− 1^ and 1063 cm^− 1^).

Trace element analysis of engine oils was conducted using a Maxine HD multi-element analyzer (XOS, USA). The analyzer employs high-resolution X-ray fluorescence (HDXRF) to quantify trace elements in hydrocarbon-based liquid samples. The device underwent calibration before measurement. The oil sample was thoroughly mixed and heated to 40 °C. Subsequently, 1 milliliter of oil was dispensed into a measuring cup using a propylene foil liner, via an automatic pipette. The analysis was performed in triplicate. The paper presents the average results.

The oil dispersion properties were assessed using a blotter spot test. A representative sample of the oil, in the form of a drop, was applied to a chromatographic paper (model SG), and the resulting spot was observed after a 24-hour drying period. The evaluation focused on the core of the spot (inner circle) and its diffusion zone (outer circle). The oil quality was assessed according to the classification proposed by motor checkup^37^. After the droplet had spread, the resulting stain was compared to an attached rating scale, which classifies the oil as good, acceptable, or critical. A poor oil quality level is determined by the following characteristics of the stain: (1) Color: Dark color may indicate the presence of contaminants or degradation products. (2) Shape and texture: Irregularities, leaks, or fringes may suggest the presence of water, fuel, or other contaminants. (3) Size of zones: The absence of distinct zones may indicate a loss of the oil’s dispersant properties.

## Results and discussion

###  Kinematic viscosity results

**Fig. 2:**
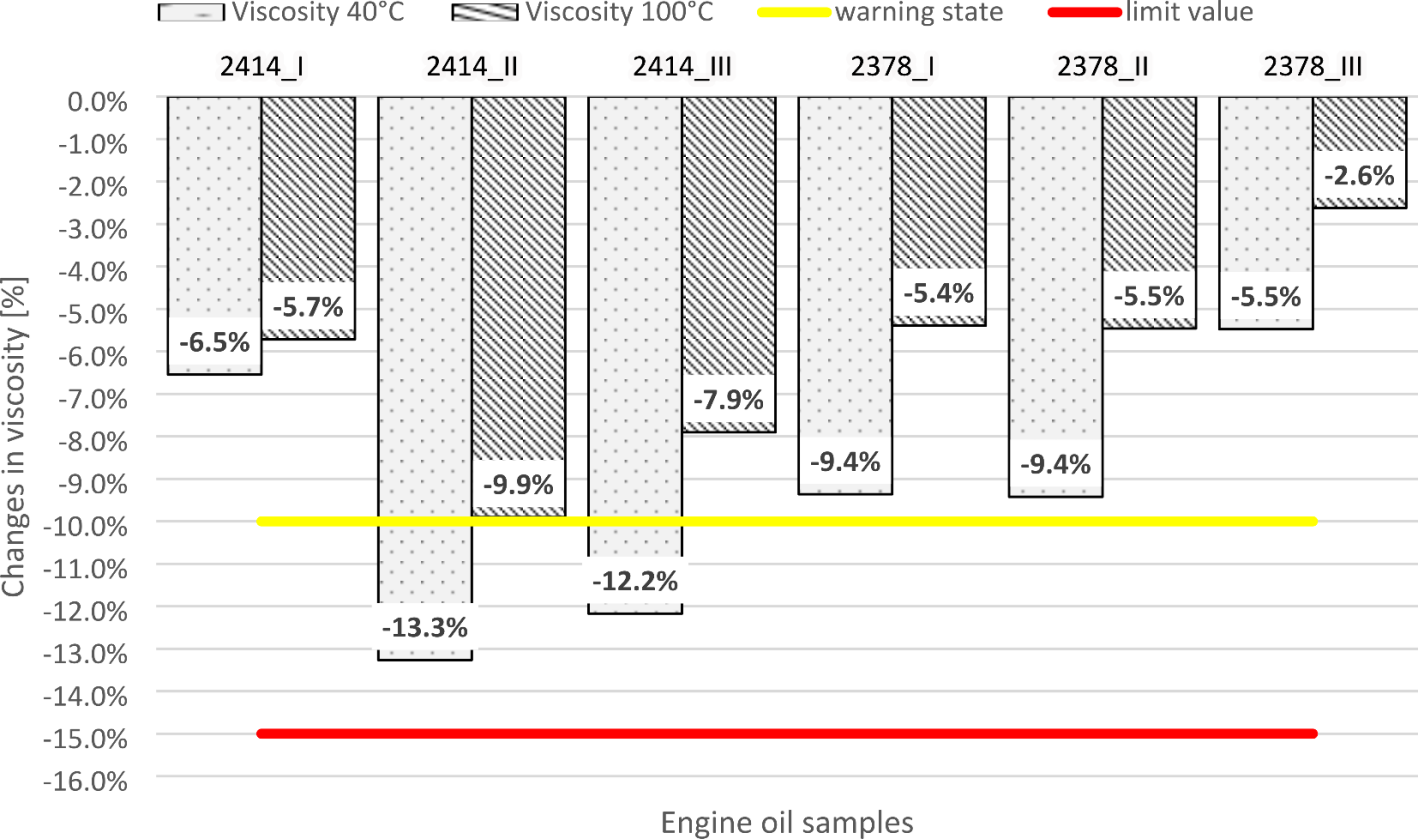
Kinematic viscosity of the tested engine oil samples.

Figure 2 shows the percentage changes in kinematic viscosity at 40 °C and 100 °C for all tested oils relative to fresh oil. The study classified changes as critical (red line) for values exceeding ± 15%, warning (yellow line) for ± 10–15%, and acceptable for less than ± 10%. The values for warning and limit states were proposed on the basis of a review of existing literature^25,38,39^.

The oil sample was tested, and the results did not exceed the established critical limit of ± 15% viscosity change, despite exceeding the manufacturer’s recommended oil change interval. Sejkorova et al.^40^, also reported no limit value exceedance for this parameter in their studies, where engine oils from buses exceeded the recommended interval by 7,500 km and 17,500 km. Their findings revealed that the kinematic viscosity values at 40 °C and 100 °C was estimated by approximately − 10% compared with those of the new oil. The oil samples from bus 2378 did not exceed the warning value in any of the tested periods. However, bus 2414 exceeded the warning value for the viscosity parameter at 40 °C twice. A comparison of these results with the failure log for bus 2414 from Table 4 reveals that the bus experienced engine overheating and damage to a unit injector. These failures, which resulted in oil dilution with unburned fuel, contributed to the observed reduced oil viscosity. For both buses 2414 and 2378, the percentage change in viscosity decreased in the final oil measurement compared with that of the fresh oil. This is most noticeable for bus 2378, with a decrease from − 9.5% to -5.5% at 40 °C and from − 5.5% to -2.6% at 100 °C. Similar trends were observed in the TBN and TAN parameters and the blotter spot test (information are visible in “Oil contamination” section, Tables 5 and 6). A comparison of these results with the information in Table 2 reveals a significant difference in the number of oil top-ups used in the last measurement period compared with the previous ones. Bus 2378 received 12 L of fresh oil in the month before the oil change, which was 54.5% of the oil pan capacity. Bus 2414 received 7 L or approximately 32% of the oil pan capacity. Over the entire analyzed oil interval, bus 2414 had 65 L of oil added to 22 L of the oil pan, whereas bus 2378 had 82 L. These large amounts of oil top-ups before the oil change periods, significantly improved the rheological properties of both tested oils.

### Oil contamination

**Fig. 3:**
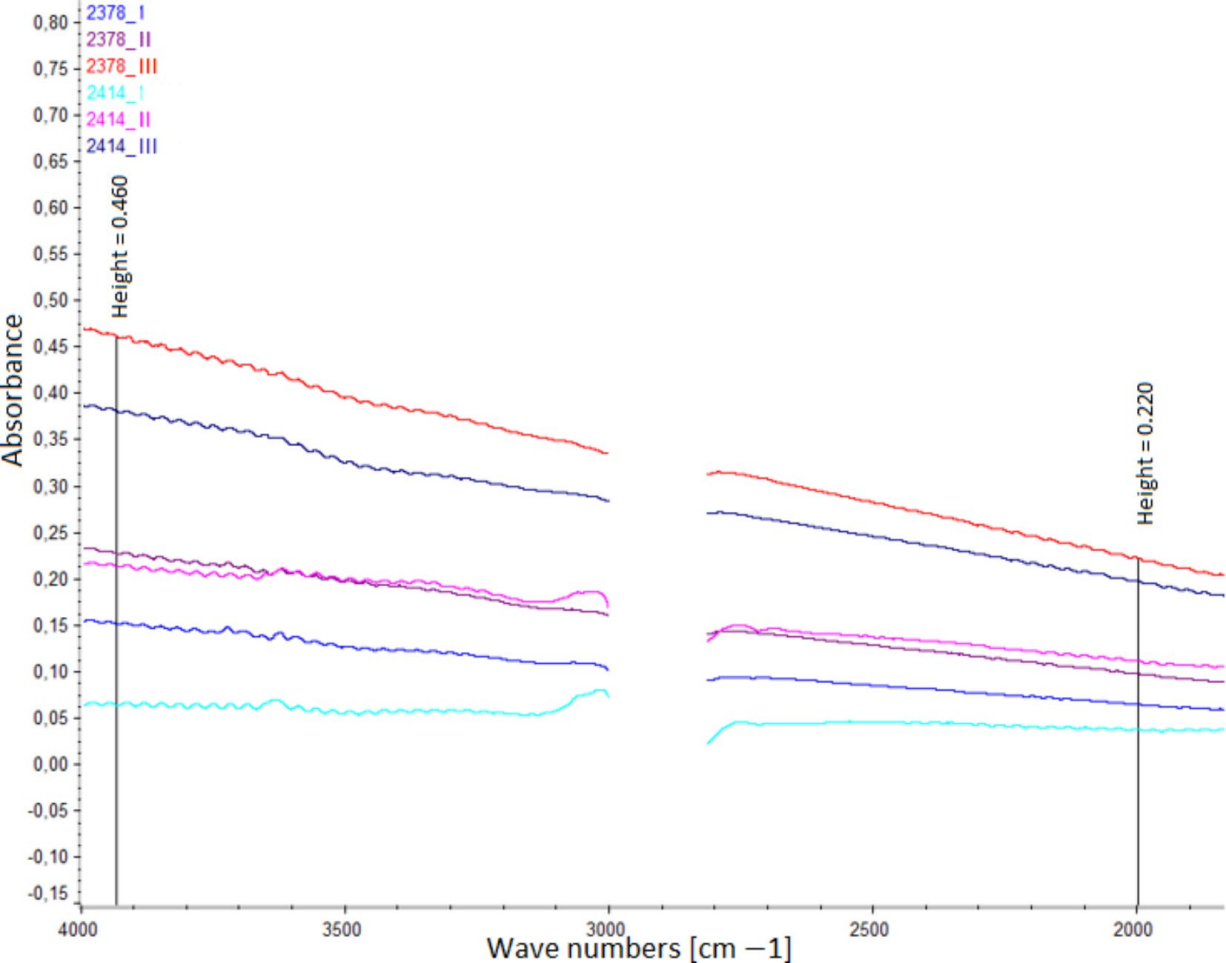
Infrared spectroscopic analysis of soot content in oil.

Infrared spectroscopic analysis for soot content was performed at two specific measurement points. Characteristic absorption bands for soot contamination were diagnosed for wavelengths of ~ 2000 cm^− 1^ and ~ 3980 cm^− 1^. Figure 3 illustrates the differentiation of the analyzed oils at the indicated points.

**Fig. 4:**
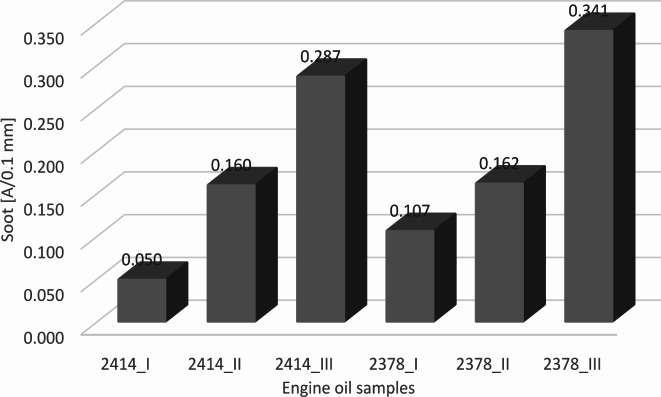
Average soot content (calculated from infrared readings at ~ 2000 cm^− 1^ and ~ 3980 cm^− 1^).

Figure 4 shows the soot content of individual oils in quantitative terms. The results of the analysis of the oil from bus 2378, with an extended oil change interval of 10,000 km, revealed the highest soot content. The average soot content (calculated from infrared readings at ~ 2000 cm ^− 1^ and ~ 3980 cm ^− 1^) was 16% higher in the oil obtained from the last sampling period in bus 2378 (0.341 [A/0.1 mm]) than in the oil from bus 2414 (0.287 [A/0.1 mm]), which also had an extended oil change interval of 6000 km. The presented soot content values are consistent with the author’s previous work^16^, where the average soot value in the analyzed city buses with a mileage of 30,000 km was approximately 0.179 [A/0.1 mm]. When comparing the soot content results with the kinematic viscosity results from the last measurement periods, it should be noted that the increased amounts of fresh oil top-ups used in this case did not significantly reduce the amount of accumulated soot in the engine. Detergents contained in the oil are more effective in removing fresh contaminants than in dissolving already deposited soot^41,42^. To remove larger amounts of soot, appropriate cleaning procedures, such as flushing the oil system, are necessary. In their 2017 study, Motamen Salehi et al.^43^ examined the correlation between oil contamination, degradation, and wear of engine oil pumps. Their findings revealed a significant relationship between the consumption of additives by soot in engine oil and accelerated wear of oil pump components during the aging process.

Table 5 presents a view of all the oil spots on chromatographic paper (read after 24 h) used to determine contamination (spot core) and dispersion properties (scattering zone) in the used oils. The figure shows a view of the spots formed after applying one and three drops of oil. The photos of the spots on the chromatographic paper were taken to the same scale, allowing direct comparison of the size and shape of the spots. The size of the spots can indicate the ability of the oil to disperse contaminants. Larger spots may indicate better dispersion of contaminants, while smaller spots may be associated with poorer dispersion. The shape of the spots provides information about the contaminants and the chemical state of the oil. A regular shape of the spot with a clear center and a diffuse zone around the outside suggests good quality oil, while irregularities such as leaks or fringes may indicate the presence of water, fuel or glycol.

**Table 5:** Characteristics of changes in engine oil dispersion properties.

Bus no	2378		
Overall mileage [km]	820,550	835,826	874,729
Mileage since last oil change [km]	2378_I 16,421	2378_II 31,697	2378_III 70,600
Blotter spot test (single drop after 24 h)			
Blotter spot test (three drops after 24 h)			
Bus no	2414		
Overall mileage [km]	653,913	675,751	704,454
Mileage since oil change [km]	2414_I 15,693	2414_II 37,535	2414_III 66,238
Blotter spot test (single drop after 24 h)			
Blotter spot test (three drops after 24 h)			

The blotter spot test and the FTIR analysis showed similar results for the soot content. Both tests revealed high levels of soot in the last sampling periods. The observations concerned the color of the core/center of the stain, the color of which may indicate the content of heavy insoluble mechanical contaminants of the oil, such as metal particles, soot, dirt, or dust. The analysis of oil droplets at the first two measurement points for bus 2414 indicates a very low-quality status according to the classification defined by the motor checkup^37^. This suggests an increased concentration of soot and other contaminants in the engine system, which requires further diagnostics. The stain from bus no. 2378 suggests a much higher quality of the oil than that from the other bus due to several key characteristics. First, the stain from bus 2378 has a more regular shape, indicating better dispersion properties of the oil and the absence of serious impurities such as water or glycol. Second, the color of the stain is lighter and less intense, suggesting less soot and oxidation products. In addition, the stain from bus 2378 does not show obvious leaks or fringes around the edges, which usually indicate the presence of water or other fluids that could degrade the oil quality. The last measurement points of both vehicles revealed a much worse condition of the oil, classifying it as critical^37^. In such a situation, an urgent service inspection is recommended. The causes of such a condition may include, among others, incorrect combustion, damage to the injection system, clogged oil filter, and problems with the exhaust system or turbocharger. The consequences of neglect can lead to soot deposits on key engine components such as valves and pistons, reduced heat transfer, accelerated engine wear, and increased fuel consumption.

The shape and color of the oil diffusion zone (outer circle) were subsequently assessed. The color of the diffusion zone is again noticeably better for the oil from bus 2378. In turn, in both cases, from both buses, there are visible frays on the diffusion zone, indicating the presence of water in the oil. These are not large amounts but are worth emphasizing. In the case of bus 2414, at measurement points 2414_I and 2414_II, a yellow halo over the central part of the stain was also clearly visible, as was a strong distortion of the outline of the stain core, which could indicate the presence of coolant in the oil. Glycol can interfere with oil dispersion, often causing a distinct, dark ring of soot surrounding the central area with a yellowish or brown hue^44,45^. Such observations can provide valuable diagnostic information when deciding on service repairs to the cooling system. Between periods 2414_I and 2414_II, the presence of the outermost semitransparent ring can also increase, indicating that fuel enters the oil. Comparing these results with the failure log presented in Tables 3 and 4, we can see some analogies in the failures that occurred, which were also visible in the blotter spot test. In the case of bus 2414, we had to address a greater number of recorded coolant leaks, and cases of fuel leaks in the bus were noted.

**Fig. 5:**
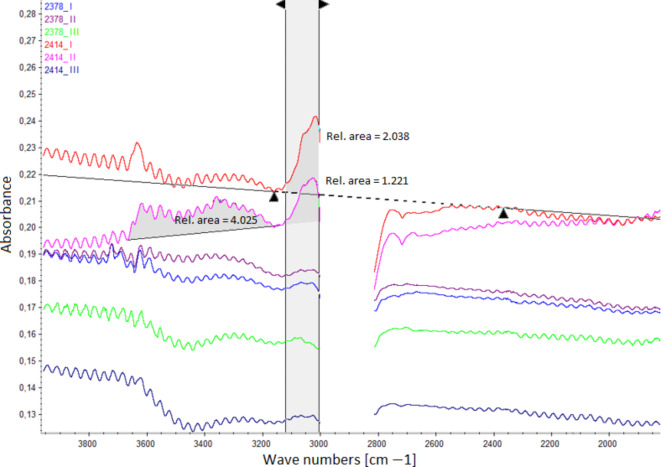
FTIR analysis of contamination levels in the tested oils.

Figure 5 shows the FTIR interferogram that shows the contaminants in the oil located between ~ 3100^− 1^ and ~ 3600 cm^− 1^ and between ~ 3000^− 1^ and ~ 3100 cm^− 1^.

In the 3000–3600 cm^−1^ range, contaminants such as water and fuel traces are evaluated. Water is particularly noticeable in this range, which may indicate water condensation in the oil or cooling system problems. In the 3000–3100 cm^−1^ range, the presence of aromatic and unsaturated hydrocarbons can be detected, suggesting possible fuel dilution of the oil. Notably, samples 2414_I and 2414_II contained relatively high levels of impurities. These findings align entirely with the results of the oil stain analysis from the blotter spot test shown in Table 5.

In addition to the previous analyses, the oils were also tested for total acid number, total base number, and glycol content. Total Base Number (TBN) is a crucial parameter that quantifies the alkaline reserve of lubricating oils. This reserve, primarily derived from overbased detergents and amine-based additives, enables the oil to neutralize acidic combustion byproducts, such as sulfur-based acids, and disperse soot particles. A higher TBN value indicates a greater capacity to mitigate acid-related wear and maintain engine cleanliness. Total Acid Number (TAN) measures the amount of acid present in oil, expressed as the milligrams of potassium hydroxide (KOH) required to neutralize one gram of oil. Monitoring TAN and TBN values optimizes oil quality and engine performance. Ethylene glycol content signals coolant contamination, which can cause severe engine or machinery damage if left unchecked. High ethylene glycol levels can lead to increased wear, corrosion, and equipment failure. The results of these tests can be found in Table 6.

**Table 6:** Total base and acid number in the analyzed oils.

No.	Sample code	Bus mileage [km]	Mileage since last oil change [km]	TBN [mg KOH/g]	TAN [mg KOH/g]	Ethyl. glycol content [wt%]
				x̅ ± SD	x̅ ± SD	x̅ ± SD
0	Fresh oil	--	--	10.57 ± 0.06	0 ± 0.00	0 ± 0.00
1	2414_I	653,913	15,693	6.10 ± 0.10	1.33 ± 0.12	0.16 ± 0.01
2	2414_II	675,751	37,535	3.90 ± 0.00	2.70 ± 0.00	0 ± 0.00
3	2414_III	704,454	66,238	8.53 ± 0.06	0.43 ± 0.06	0.25 ± 0.01
4	2378_I	820,550	16,421	8.87 ± 0.06	0.63 ± 0.06	0.32 ± 0.00
5	2378_II	835,826	31,697	8.73 ± 0.06	0.67 ± 0.06	0.40 ± 0.01
6	2378_III	874,729	70,600	7.90 ± 0.10	1.10 ± 0.00	0.53 ± 0.01

x̅ - arithmetic average, SD – standard deviation.

Analyzing the total base number results, the most significant decrease in neutralization properties was observed in sample 2414_II, with a 63% reduction compared with that of fresh oil. Similarly, a large decrease of 42% was noted in sample 2414_I during the previous sampling period. Such decreases are often attributed to increased contaminants such as soot, water, or elevated combustion chamber temperatures, which promote the formation of acidic compounds. Additionally, coolant contamination can clog oil filters, leading to reduced oil flow, potential filter bypass, and compromised overall oil efficiency. The oil in bus 2378 maintained significantly better neutralization properties throughout the oil interval. When these observations are compared with the failure records in Tables 3 and 4, we again observe a correlation between the detected faults and the oil analysis results. Both buses experienced cooling system failures, which were indicated in the oil analysis by the presence of coolant components, specifically glycol. For bus 2378, the glycol content in the engine oil increased with each subsequent oil measurement. Common causes of such contamination include a cracked head gasket, a damaged water pump, or faulty pipes or connections^44,46^. Visually diagnosing coolant-oil mixing can sometimes be done by observing a mayonnaise-like white color of the engine oil. Other signs include coolant foaming, a drop in coolant level in the expansion tank, and engine overheating.

**Fig. 6:**
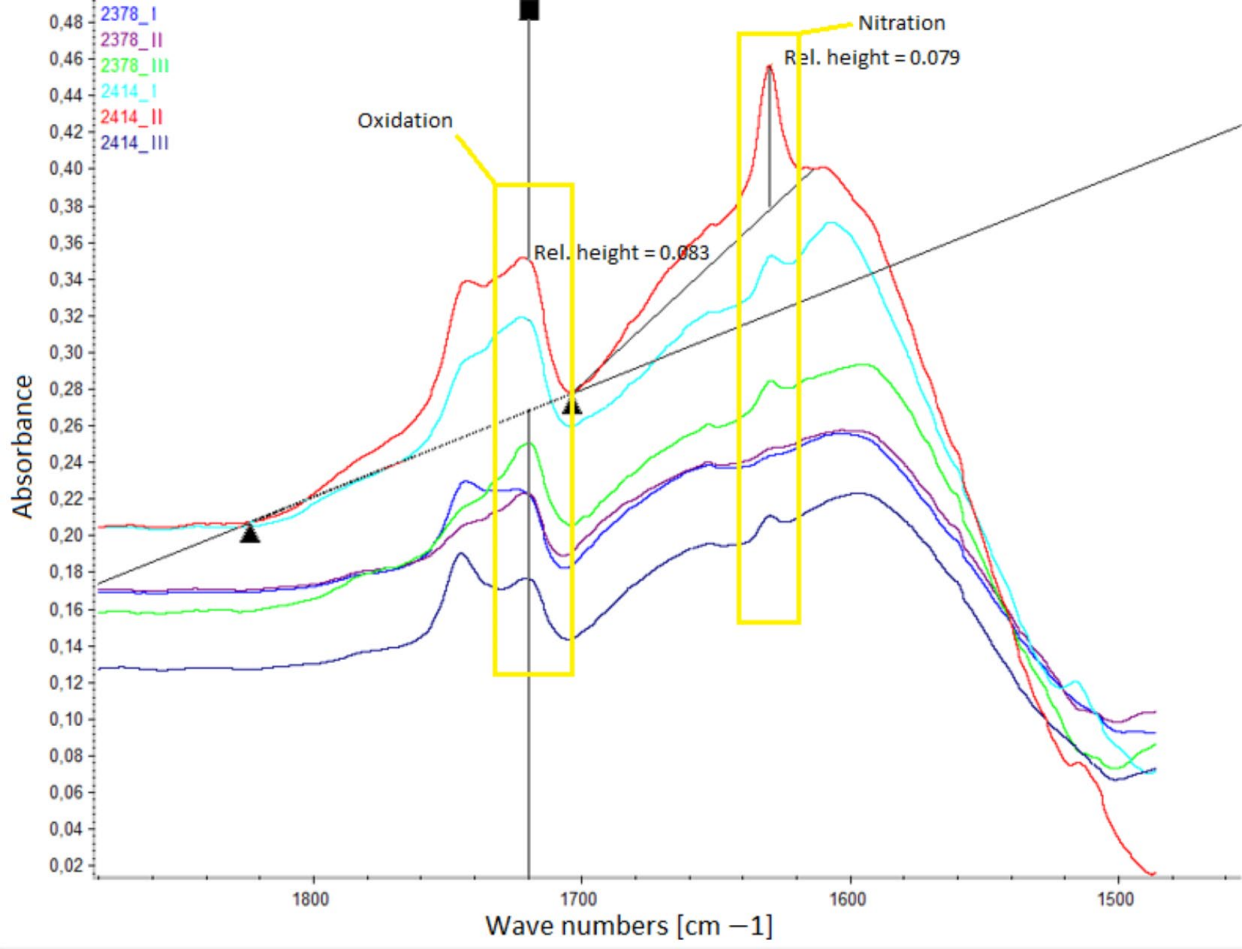
FTIR spectrum analysis of nitration and oxidation in the tested oils.

Figure 6 shows the FTIR spectra related to nitration (approximately ~ 1630 cm^− 1^) and oxidation (approximately ~ 1720 cm^− 1^).

FTIR spectra analysis (Fig. 6) revealed that oil sample 2414_II presented the highest intensity of absorption bands in regions indicative of nitration (approximately ~ 1630 cm ^− 1^) and oxidation (approximately ~ 1720 cm^− 1^). Referring to the failure log in Table 4, the most significant issues during this period were related to the cooling system. Such failures can lead to engine overheating, which increases combustion chamber temperatures, promoting more intense chemical reactions, including oxidation and nitration. The absorbance values for these bands were 0.083 and 0.079 A/0.1 mm, respectively, suggesting an advanced stage of oil degradation due to oxidation processes. These observations align with the total base number (TBN) and total acid number (TAN) analysis results. Notably, similar to kinematic viscosity, FTIR spectra analysis revealed a surprising result: sample 2414_III, theoretically the most used oil, presented better TAN and TBN parameters than earlier samples did. A comparison with samples from bus 2378 further highlights this discrepancy. This result may indicate that the last oil sample was taken after a significant amount of fresh oil was replenished, effectively refreshing the oil and improving its quality.

##  Elemental analysis

Table 7 presents the results of the elemental analysis of the tested oils in terms of the amount of the engine wear and the elements responsible for additives.

**Table 7:** Characteristics of changes in engine wear and the consumption of additives in used engine oils.

Sample data	Engine wear [ppm]				Additives [ppm]				
Sample code	Cr	Cu	Fe	Ni	Ca	Mo	*P*	S	Zn
	x̅ ± SD	x̅ ± SD	x̅ ± SD	x̅ ± SD	x̅ ± SD	x̅ ± SD	x̅ ± SD	x̅ ± SD	x̅ ± SD
New oil	0.39 ± 0.18	--	1.27 ± 0.03	0.17 ± 0.03	4305 ± 14.74	379 ± 23.58	960 ± 30.89	3806 ± 14.15	1376 ± 3.21
2414_I	0.84 ± 0.08	5.83 ± 0.03	28 ± 0.46	0.34 ± 0.04	3126 ± 26.50	391 ± 18.23	671 ± 41.58	4181 ± 20.00	1049 ± 2.00
2414_II	2.26 ± 0.10	7.23 ± 0.12	40 ± 0.12	0.64 ± 0.08	2851 ± 16.92	383 ± 9.45	686 ± 32.79	3349 ± 7.64	961 ± 0.58
2414_III	0.99 ± 0.04	2.14 ± 0.14	76 ± 0.58	0.39 ± 0.05	4614 ± 16.64	387 ± 11.53	907 ± 14.11	3594 ± 23.46	1405 ± 0.58
2378_I	0.76 ± 0.05	274 ± 0.00	26 ± 0.34	0.22 ± 0.14	4022 ± 12.50	365 ± 7.57	827 ± 11.72	3290 ± 15.01	1275 ± 0.58
2378_II	0.77 ± 0.03	76 ± 0.11	25 ± 0.14	0.28 ± 0.05	4508 ± 42.34	351 ± 9.54	936 ± 27.40	3411 ± 23.50	1387 ± 0.58
2378_III	1.61 ± 0.04	11.3 ± 0.10	63 ± 0.83	0.80 ± 0.05	5072 ± 36.06	366 ± 9.45	1001 ± 13.32	3616 ± 8.89	1544 ± 5.51

x̅ - arithmetic average, SD – standard deviation.

Notably, the copper contents in samples 2378_I and 2378_II were very high at 274 ppm and 76 ppm, respectively. Sejkorova et al. reported similar copper contents in city buses, with an average of 8.13 ppm compared with bus 2414 presented in this study. Zachar et al.^47^ investigated the effect of electron beam irradiation of engine oil on its performance properties. In samples from a passenger car, the copper content at the time of engine oil change at 16,000 km was 7 ppm, whereas in the previously irradiated oil sample, it was 5.5 ppm. According to Aucélio et al.^48^, copper particles in oil appear as a result of the wear of valve guides, piston rings, and support points. Table 3 shows that this type of situation is related to the recorded failure caused by a faulty cooling system and leaks of coolant (glycol) into the fuel. Zhu et al.^49^ emphasized the corrosive effects of coolant contamination on oil, especially on copper surfaces.

The presence of iron in engine oil is one of the most frequent and most alarming signals indicating the wear of mechanical engine components^50,51^. In this case, it is worth noting the very large difference in iron content between the second and third measurement points. The presence of iron in the oil is a result of the wear of the most important engine components, including cylinders, pistons, gears, rings, axles, oil pumps, crankshafts, and support bearings. In the case of bus number 2414, this increase reached 47%, while in the case of bus 2378, it was a 60% difference. Due to this, it seems reasonable to change the oil in both cases after 30,000 km.

The content of other metallic compounds, including Cr, and Ni, was negligible compared to the previously discussed elements related to engine wear. However, when considering the percentage increase in these elements compared to fresh oil, a different perspective emerges. Chromium, a common material used as a coating to enhance wear resistance and reduce friction, can be found in various mechanical components of internal combustion engines, including piston rings, cylinder liners, and valve train components^52^. The chromium content of sample 2414_II exhibited the largest percentage increase, exceeding that of the new oil by more than four times. In^53^ the authors investigated the impact of various field operations on the wear of agricultural tractor engines. The chromium content in the cultivator, which exerted the greatest engine effort, was 0.6 ppm. Zając et al.^54^ analyzed the amount of engine wear in passenger cars and reported that the highest value of this element in diesel engines was 8.1 ppm, which was 72% higher than the maximum value observed in the tested buses. Higher chromium values were also reported in^55,56^.

Regarding nickel content, the largest percentage difference compared to new oil, nearly four times, was observed in the final oil sampling period of bus 2378. In the preceding periods, these differences fluctuated, reaching 29% in the first period and 65% in the second. For both tested buses, the absolute values remained below 1 ppm. Similar nickel contents were reported in the studies^53,54,57^. Stout et al.^55^ analyzed over 150 oil samples, finding an average nickel content of 2.5 ppm and a maximum recorded value of 15 ppm.

Analyzing the changes in the depletion of additives, once again, after the kinematic viscosity parameter and the degree of oxidation, we can observe the effect of its refreshment at the last measuring point before the change. Moreover, due to the regeneration of the engine head in bus 2378 at the beginning of the oil interval, the changes in the depletion of additives were at a lower level compared to bus number 2414. The content of sulfur, which comes from various additives used to enhance performance properties such as antiwear (AW), extreme pressure (EP) as well as antioxidants^58^, in the first two measuring points in the cases of both vehicles, exceeded a 10% decrease in relation to fresh oil. However, at the measuring point representing the moment of oil change, this difference decreased to 5% in both cases. It should be noted in this situation that the large refills of fresh oil used did not affect the reduction of iron content, which at the time of oil change oscillated at its limit values.

Calcium in engine oil serves as an additive responsible for detergent and dispersant properties^59,60^. Additionally, calcium is frequently a component of coolants, particularly those based on organic carboxylic acids (OAT). Consequently, the presence of calcium in oil may signal coolant leaks and engine issues. Examining the final measurement points in the analyzed buses and considering the oil refreshment effect observed during this period, significant increases in calcium levels are apparent. This could indicate potential problems with the cooling system and necessitate immediate attention.

Zinc, though not directly incorporated, is an essential element in Zinc Dialkyldithiophosphate (ZDDP), a prevalent additive in lubricants^61^. ZDDP is a multifunctional oil additive that provides anti-corrosion protection, reduces friction, and keeps the engine clean. An increase in zinc content in engine oil is usually a sign of wear or damage to protective coatings of specific engine components, such as plain bearings, bearing shells, pistons, or camshafts. Additionally, looking at the last measurement points in the cases of both vehicles, instead of depleting the additives, we can see the effects of wear already taking place. This is another signal indicating the need to reduce the duration of the engine oil interval to protect the engine from excessive wear.

## Conclusions

In order to ensure long-term engine operation, regular oil quality checks are necessary. Each failure, from oil, fuel, or coolant leaks to injector damage or overheating, causes noticeable changes in oil parameters.

In summary, the analyses of oil parameter changes in engines with diagnosed faults yielded the following conclusions. The first potential source of information about a cooling system failure may be the presence of coolant or water in the oil. The results of the conducted FTIR spectrometric analysis, as well as the blotter spot test, suggest the potential presence of these contaminants in the oil. However, elemental analysis using X-ray fluorescence did not detect elements such as sodium, boron, or potassium, which are commonly used in coolant additives^45^.

A second approach to identifying failure causes was to analyze changes resulting from oil overheating. Both buses experienced engine overheating, which likely affected the oil. FTIR analysis revealed a correlation between recorded failures related to increased engine operating temperature and accelerated oxidation. The key question is how to connect the cause (engine overheating) to the effect (oil oxidation) in the context of engine failure prediction. Based on the conducted tests, assessing the degree of oxidation may not reliably predict imminent engine overheating and subsequent failure. However, it is important to note that this type of research is not intended for immediate predictions. The focus is on estimating the probability of failure based on oil parameters. Therefore, regular oil sampling at more frequent intervals is recommended to provide early warnings of potential issues.

The authors also highlight a significant issue related to drawing conclusions about oil condition: the impact of oil top-ups. Throughout the analyzed oil interval, bus number 2414 received 65 L of oil added to the original 22 L of oil pan, while bus 2378 received 82 L. Frequent oil top-ups, significantly exceeding the oil pan’s capacity, introduce substantial disruptions in the oil condition monitoring process. Regular top-ups can have a “refreshing” effect on the sample, leading to lower oil degradation parameter values and hindering accurate assessment of its actual condition. While top-ups reduced the oil viscosity parameter’s degradation in the final measurement period, they had a less significant impact on reducing soot content. Consequently, the obtained results may not accurately reflect the actual degree of oil consumption, potentially leading to misinterpretations of the engine’s technical condition. One potential solution is to frequently analyze results just before planned oil top-ups. Alternatively, more frequent use of simple blotter spot tests can help diagnose trends in engine oil parameter changes. Although blotter spot tests offer limited accuracy and provide only a qualitative assessment of oil condition, they can be useful for monitoring changes. They do not allow for precise determination of contaminant concentrations or the degree of oil degradation, nor do they provide information on other important parameters like acidity or oxidation state. Additionally, the interpretation of test results can be subjective and depend on the experience of the person conducting the test.

The data from the blotter spot test were consistent with the oil parameters measured via the FTIR and XRF methods, indicating a high level of compliance. Analysis of the oil stain on chromatographic paper effectively differentiated the samples on the basis of impurity content (soot, water, and glycol, without definitive assignment). This information is especially valuable in diagnosing critical cooling system failures. The low cost and high effectiveness of blotter spot tests, as confirmed by the authors, make them suitable for inclusion in routine vehicle maintenance. Frequent use of these tests can provide a general assessment of both oil and engine conditions, potentially leading to early detection of potential engine failures.

### Limitations of the study

The limited sample size is a significant limitation when generalizing the results to a larger population. This study was a pilot study and was used to initially identify potential correlations between the oil condition and technical condition of the vehicle in real operating conditions.

Despite the limited number of vehicles tested, the obtained results provided valuable premises that can be the basis for further, more extensive research. The main goal was to examine the possibility of predicting failures based on the analysis of oil parameters. However, to confirm these initial conclusions, it is necessary to conduct research on a larger fleet of vehicles over a longer period of time.

Expanding the scope of the research will increase the statistical significance of the results obtained, and thus strengthen the credibility of the conclusions formulated. This will allow for the development of more accurate tools for predicting failures based on oil analysis.

## Data Availability

The data presented in this study are available on request from the corresponding author.
